# Evaluation of Ankle Fractures in 228 Patients From a Single Center Using Three-Dimensional Computed Tomography Mapping

**DOI:** 10.3389/fbioe.2022.855114

**Published:** 2022-03-15

**Authors:** Jianshuang Zeng, Cheng Xu, Gaoxiang Xu, Wupeng Zhang, Daofeng Wang, Hua Li, Xuewen Gan, Ying Xiong, Jiantao Li, Licheng Zhang, Peifu Tang

**Affiliations:** ^1^ School of Medicine, Nankai University, Tianjin, China; ^2^ Senior Department of Orthopedics, The Fourth Medical Center of PLA General Hospital, Beijing, China; ^3^ National Clinical Research Center for Orthopedics, Sports Medicine and Rehabilitation, Beijing, China; ^4^ Department of Orthopedics, Yanan Hospital, Kunming Medical University, Kunming, China

**Keywords:** ankle fracture, LH classification, AO classification, 3D mapping, 3DCT

## Abstract

**Purpose:** The ankle joint has a complex anatomy structure with many causative factors and various injury mechanisms, and the clinical presentation of ankle fractures is diverse. This study aimed to analyze the characteristics of ankle fractures by applicating three-dimensional fracture line mapping technique.

**Methods:** A retrospective study was conducted on 228 patients with ankle fractures. Three-dimensional reconstruction was performed by CT images and the fracture reconstruction model was superimposed onto a standard model of the tibiofibula for fracture line drawing. Then the fracture lines were converted into a three-dimensional coordinate point data set. And the fracture line maps as well as the fracture line heat maps were generated in 3-Matic software and Unigraphics NX software, respectively.

**Results:** The dense area of the fibular fracture lines was located above the tibiofibular joint ligament and wrapped obliquely around the distal fibula from the anterior edge of the fibular neck. The fibular fracture line could be divided into three categories according to the degree of denseness. The dense area of the tibial fracture line is located within the anterior tibial fornix, the anterolateral corner, and the fibular notch. The tibial fracture lines can be classified into four categories according to the density of the fracture lines. The combined medial malleolus + posterior malleolar fracture line situation was found to be not encompassed by the existing AO and Lauge-Hansen (LH) classification systems according to this classification.

**Conclusion:** The 3D fracture line mapping technique can better reflect the distribution of ankle fracture lines and could help to establish a new ankle fracture typing system in the future.

## Introduction

Ankle fractures account for approximately 3.9% of total body fractures (Koval et al., 2005), of which the incidence has a tendency to increase year by year with the aging process of the population and social development. Since the complex anatomical structure of the ankle joint, the various causative factors, and the mechanism of injury containing both vertical extensive force at a strong level and rotational extensive force at a moderate level, ankle fractures are clinically presented in various forms. How to comprehensively and accurately assess ankle fractures, understand fracture injury mechanisms and select appropriate treatment methods are also very important in clinical practice. As the main method to describe the fracture injury pattern, fracture classification is the most scientific, simple, and practical method to analyze the fracture injury mechanism, guide the treatment, and judge the prognosis, which plays a vital role in clinical practice. However, the existing common classification of ankle fractures, the LH and AO classification, are based on plain film diagnosis. However, with the popularization of CT and three-dimensional (3D) CT technology in recent years, the research on ankle fractures in the industry has deepened, and the understanding of fracture morphology, fracture line distribution, fracture pattern, and fracture injury mechanism has become more advanced. In 2000, Hanse reported a special type of posterior fracture of the inferior articular surface of the tibia, which was named “Posterior Pilon fracture” (Sigvaard and Hansen, 2000). The fracture line was distributed in the coronal plane of the posterior malleolus and extended to the anterior medial malleolus. Unlike the traditional posterior malleolus fracture, the fracture fragment was much bigger and proximally displaced, and the articular surface was collapsed without comminuted fracture fragment, which cannot be explained by the existing ankle fracture classification.

Other findings suggest that articular surface collapse is an important factor affecting the prognosis of ankle fractures (Berkes et al., 2013; Hoogendoorn, 2017), and Jan Bartoníček et al. (Bartoníček et al., 2017) summarized the factors related to posterior malleolus prognosis and concluded that there was a significant correlation between bone mass size, articular surface flatness, and ankle stability and the choice of treatment modality and treatment outcome.

However, none of the above is captured by the existing classification system. The 3D fracture line mapping and heatmap is a new technique developed in recent years (Cole et al., 2013; Yang et al., 2018), which can clearly and visually display the distribution and frequency of fracture lines. Nowadays, it has been widely used in various fracture sites, but currently, the studies on ankle fracture are only limited to one part of the ankle fracture (lateral, medial, or posterior malleolar) (Yun et al., 2016; Blom et al., 2019; Peng, 2019; Yu, 2019; Yu et al., 2021), and there is no relevant study for the whole ankle fracture line. Therefore, this study aims to investigate the characteristics of the overall fracture line distribution in ankle fractures by using 3D CT imaging combined with heatmap techniques to explore the pattern of ankle fractures.

## Materials and Methods

### Study Eligibility Criteria

Inclusion criteria: 1). Patients aged >16 years; 2). With closed ankle fractures; 3). With preoperative CT images from our institution and the CT scan data is qualified for 3D reconstruction.

Exclusion criteria: 1). Patients with pathological fractures; 2). With open fractures; 3). With Pilon fractures and cumulative tibiofibular fractures of the ankle joint; 4). With a previous history of ipsilateral ankle deformity.

### CT Technique

All Computed Tomography (CT) examinations were performed using conventional cross-sectional scans and performed on 16-detector row CT scanners (United Imaging uCT 510 [Shanghai, China]). Scanning conditions were as follows: voltage 120 kV, current 150 mA, pitch 0.8–0.9 mm, layer thickness 3 mm, and reconstruction matrix 512 × 512. The scan area was as follows: distal tibiofibular includes ankle fracture line to mid-metatarsal segment, scan plane parallelling to the cross-section of the tibia.

### Fracture Mapping

**FIGURE 1:**
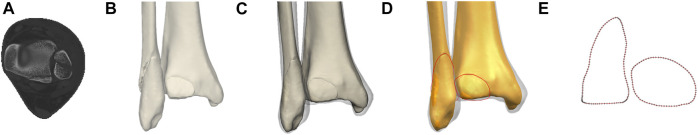
The method used for the mapping of ankle fracture. **(A)** CT image of ankle fracture. **(B)** Major fragments were reconstructed in Mimics software. **(C)** Virtual reduction and registeration with the transparent standard tibiofibular template in 3-matic software. **(D)** Delineation of fracture lines on the template in Unigraphics NX software. **(E)** Dataset extraction of fracture curves in AutoCAD software at 0.1 mm spacing equidistant.

(1) DICOM (Initial Digital Imaging and Communications in Medicine) data of a healthy male adult have been imported into Mimics 21.0 software (Materialise, Leuven, Belgium) for 3D reconstruction, keeping the intact tibial and fibular as standard templates.
(2) The DICOM data of the selected 228 patients were imported into Mimics 21.0 software (Materialise, Leuven, Belgium) for 3D reconstruction and virtual reduction of the ankle fracture fragment.
(3) Then the registeration between 3D reconstructed fracture model and the standard tibiofibular template being aligned was conducted in 3-Matic 13.0 (Materialise, Leuven, Belgium). The alignment process including rotation, translation and scale adjustment in five fields of view (anterior, posterior, internal, external and inferior tibiofibular articular surfaces).
(4) After the registration, the 3D reconstructed fracture model was imported into Unigraphics NX 12.0 (Siemens PLM Software, Co., Ltd., Plano, TX, United States) together with the tibiofibular standard template, and then the 3D fracture lines were delinated on the skeletal standard template and exported in iges format, grouped according to different fracture classifications.
(5) The same classification of fractures curves were imported into 3-Matic software and overlaid on the tibiofibular standard template to obtain the 3D fracture line distribution map.
(6) Fracture lines dataset was extracted from 3D fracture curves in AutoCAD 2020 (Autodesk Inc., San Rafael, CA, United States) at 0.1 mm spacing equidistant in the form of (x, y, z).
(7) The dataset obtained in step 6 was then imported into Originlab 9.0 (OriginLab, Hampton, MA, United States) to produce and generate heatmap of the fracture line distribution (Figure 1).

### Data Analysis

A descriptive study was performed to analyze the fracture patterns of patients. The general information was summarized as means ± standard deviations for continuous variables and as frequencies and percentages for categorical variables. The fracture maps illustrated the fracture location, while the heatmap illustrated the spatial differentiation of the fracture lines.

## Results

**FIGURE 2:**
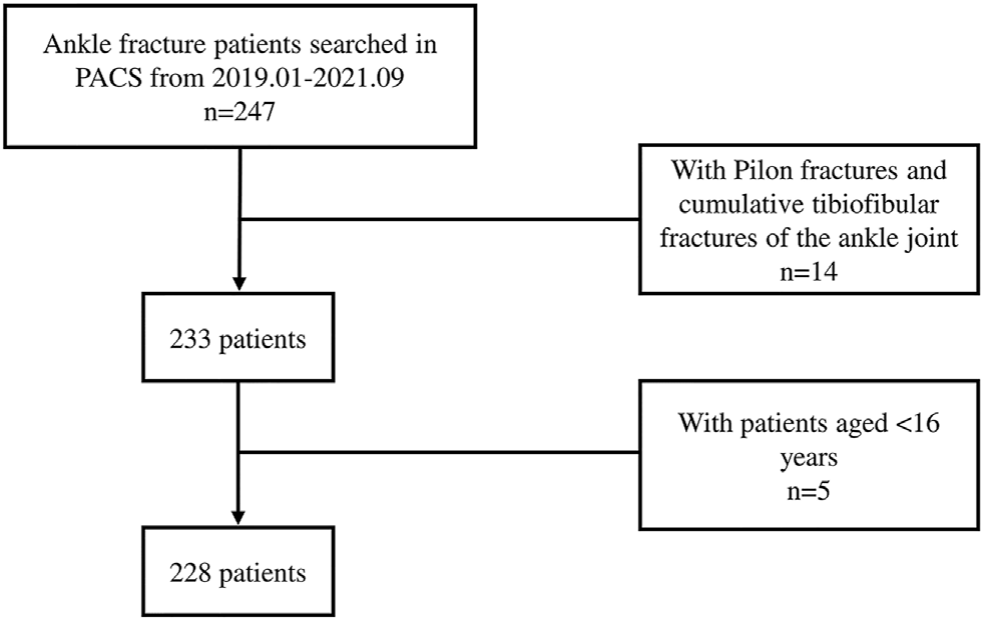
Flowchart illustrates the process of patient inclusion. PACS, Picture Archiving and Communication Systems.

A total of 228 ankle fracture patients (from 2019.01-2021.09) were retrieved from the PACS (Picture Archiving and Communication Systems) of Chinese PLA General Hospital (Figure 2). General information and fracture characteristics were summarized in Table 1. The cohort of 228 patients (including 127 men, 101 women) had a wide range of ankle fracture types. The highest percentage of fracture type was supination-external/eversion rotation (SER) with 67% in the LH classification, the least was pronation-external rotation (PA) with only 6 patients, and the number of cases of type supination-adduction (SA) and pronation-abduction (PER) was close to 8% (17 cases) and 17% (39 cases), respectively. The highest percentage of type SER was stage IV with an overall percentage of about 33%. The highest percentage of the AO classification was type B with a high percentage of 66%. The proportion of B1, B2, and B3 was close to 21,8 and 37%, respectively, where the proportion of types A and C was close to 14 and 11%, respectively.

**TABLE 1:** Patient characteristics and fracture patterns organized by two primary categories of fracture.

Character	Data (*n* = 228)	Character	Data (*n* = 228)
Female	101	AO classification	
Male	127	A1	6 (3%)
Age	42.6 years (16–87)	A2	13 (6%)
16–40y	42%	A3	11 (5%)
41–70y	51%	B1	49 (21%)
71–above	7%	B2	19 (8%)
LH classification		B3	85 (37%)
SER1	0	C1	13 (6%)
SER2	49 (21%)	C2	10 (4%)
SER3	30 (13%)	C3	3 (1%)
SER4	76 (33%)	Unspecified fracture lines	
SA1	6 (3%)	LH	10 (4%)
SA2	11 (5%)	AO	19 (8%)
PER1	16 (7%)		
PER2	2 (1%)		
PER3	5 (2%)		
PER4	16 (7%)		
PA1	0		
PA2	0		
PA3	6 (3%)		

* LH, Lauge-Hansen; SER, supination-external/eversion rotation; SA, supination-adduction; PA, pronation-abduction; PER, pronation-external rotation; AO, AO/OTA classification.

### 3D Mapping of Tibia Fracture Lines

Front view: The tibial fracture lines were mainly distributed on the lateral surface of the medial malleolus and the anteromedial corner of the tibial fornix. On the heatmap, the hot spots were also on the lateral surface of the medial malleolar and the anteromedial corner of the tibial fornix.

Back view: The fracture lines were distributed around the Volkmann’s tuberosity and on the medial malleolar surface, and the hot spot was located at the anteromedial corner of the tibial fornix.

Left view: The fracture lines extended from the anteromedial corner of the tibial fornix to the anterolateral corner of the tibial fornix and from the medial malleolar surface to the tibial fornix. The hot spot was located at the anterolateral corner of the tibial vault.

Right view: The fracture lines were distributed along the fibular notch toward the subtalar articular surface.

**FIGURE 3:**
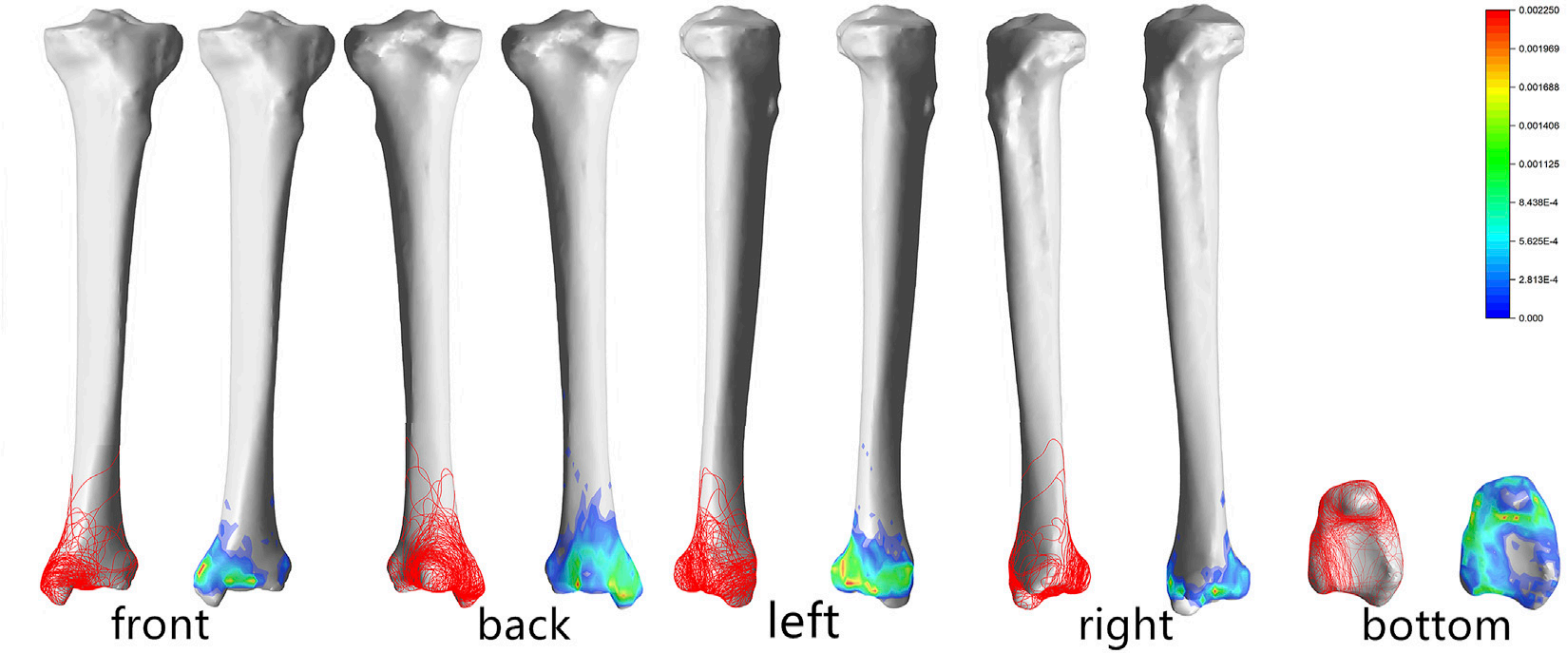
Distribution and hotpots of fractures on tibia: the front view, the back view, the left view, the right view and the bottom view (from left to right). The scale of heatmap represents relative frequency of fracture lines. From the blue area to the red area, the relative frequency gradually increases.

Bottom view: A portion of the fracture lines were distributed mainly at the interface between the medial malleolar facet and the subtalar articular facet, while another portion of the fracture line extended downward from the medial sulcus and along the medial edge of the subtalar articular facet. The lesion was located at the lateral edge of the distal lower tibial articular surface (Figure 3).

### 3D Mapping of Fibula Fracture Lines

**FIGURE 4:**
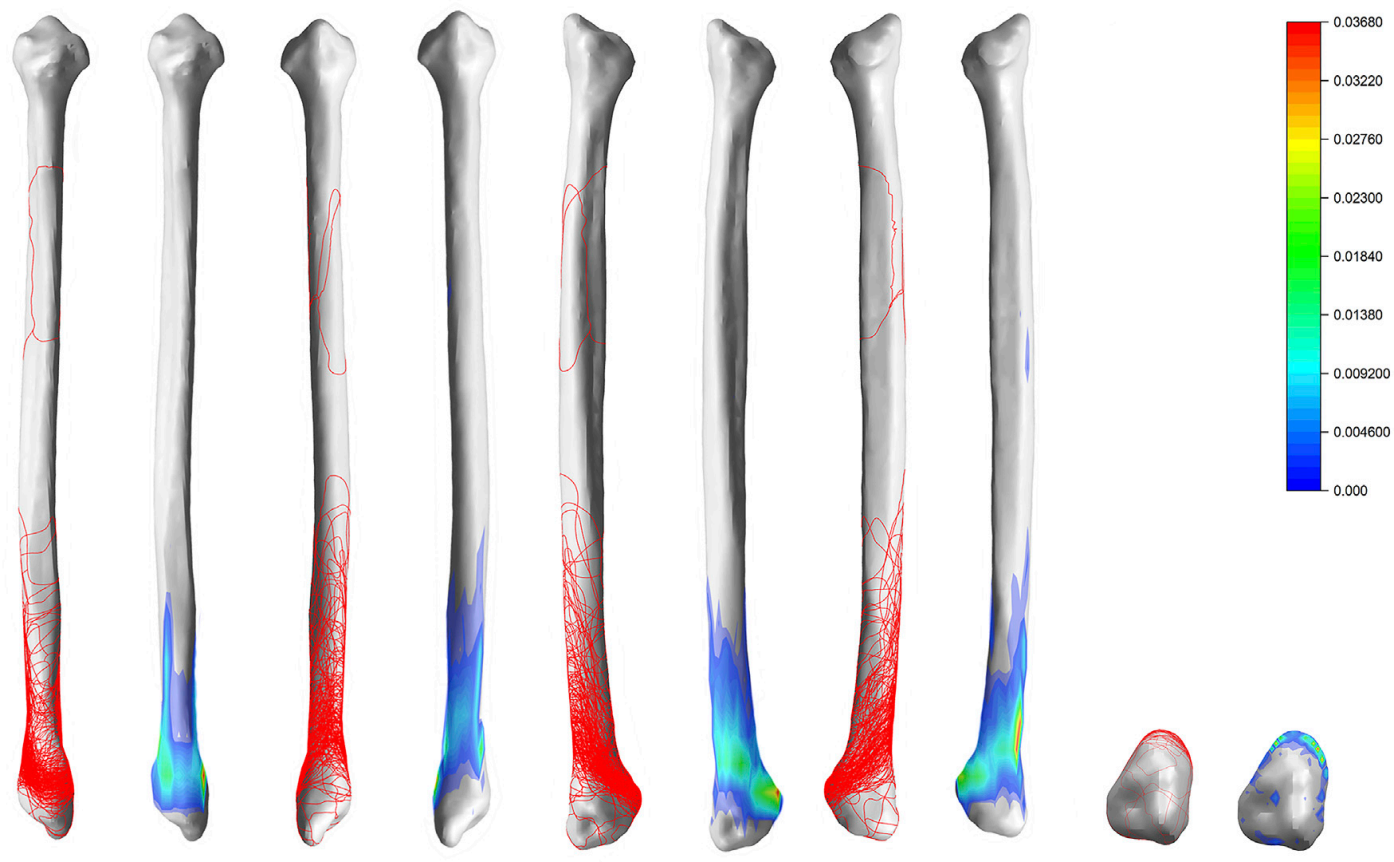
Distribution and hotpots of fractures on fibula: the front view, the back view, the left view, the right view and the bottom view (from left to right). The scale of heatmap represents relative frequency of fracture lines. From the blue area to the red area, the relative frequency gradually increases.

The fracture lines of the fibula were mainly distributed in a circular pattern, basically symmetrically along with the lower projection of the fibular neck, and traveled obliquely posteriorly past the most pronounced projection of the fibular head to converge just above the posterior edge of the fibula (Figure 4).

### 3D Mapping of L-H Classification

SER: Stage II fracture line is distributed obliquely near the fibular neck. Stage III adds a fracture line wrapped around Volkmann’s tuberosity to stage II. The hot spot on stage III is mainly located at the inferior edge of Volkmann’s tuberosity. Stage IV adds a circumferential fracture line around the medial malleolar on the basis of stage III.

SA: Stage I fracture line is mainly located on the lower part of the fibular head, and stage II fracture line is mainly located on the surface of the medial malleolar.

PA: Stage I fracture line is mainly distributed in the medial malleolar and is circular through the anteromedial and anterolateral angles of the tibial fornix and the medial surface of the ankle joint. Stage II fracture line is mainly located in the fibular stem. Stage IV mainly crosses the fibular stem and surrounds the Volkmann’s tuberosity and the medial malleolar.

**FIGURE 5:**
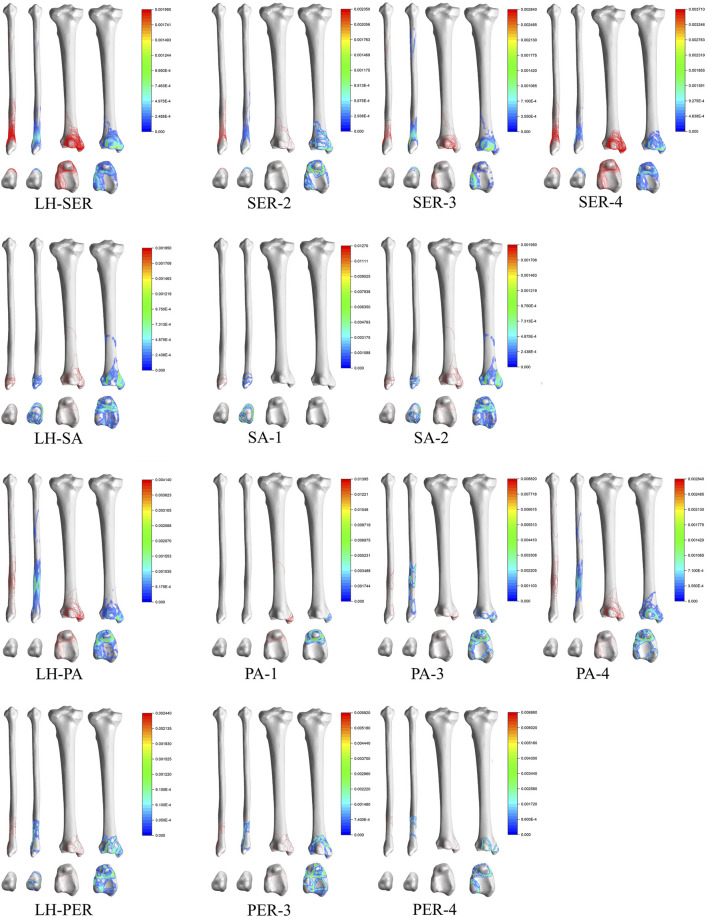
Distribution and hotpots of fractures by Lauge-Hansen classification. The scale of heatmap represents relative frequency of fracture lines. From the blue area to the red area, the relative frequency gradually increases.

PER: There were 0 cases of stage I and stage II in this study. There were only 6 cases of stage III with fracture lines distributed distal to the fibular stem (above the tibiofibular joint ligament), and there was no significant regularity in the distribution of stage III. Stage IV fractures were characterized by a circumferential fracture line extending above the tibiofibular joint and encircling the posterior and medial malleolar (Figure 5).

### 3D Mapping of AO Classification

Type A: Type A1 fracture lines were mainly distributed in the lower part of the fibular head, under the tibiofibular joint ligament, and pass through the enlarged part of the fibular head and surround it in a circular pattern. Type A2 added a circular fracture line around the medial malleolar on the basis of Type A1, with hot spots located mainly on the medial aspect of the medial malleolar, the anteromedial corner of the tibial fornix, and the prominent area below the fibular joint fossa. Type A3 fracture lines were distributed in the lower part of the fibular head, posterior malleolar, and medial malleolar, but no obvious pattern was seen on the heatmap.

Type B: Type B1 fracture lines were obliquely distributed over the tibiofibular joint ligament and around the Volkmann’s tuberosity and the medial malleolar. Type B2 was similar to Type B1 in that the tibial fracture line was distributed along the medial edge of the medial malleolar and wrapped around the medial malleolar. Type B3 is similar to Type B2 but is more densely distributed.

**FIGURE 6:**
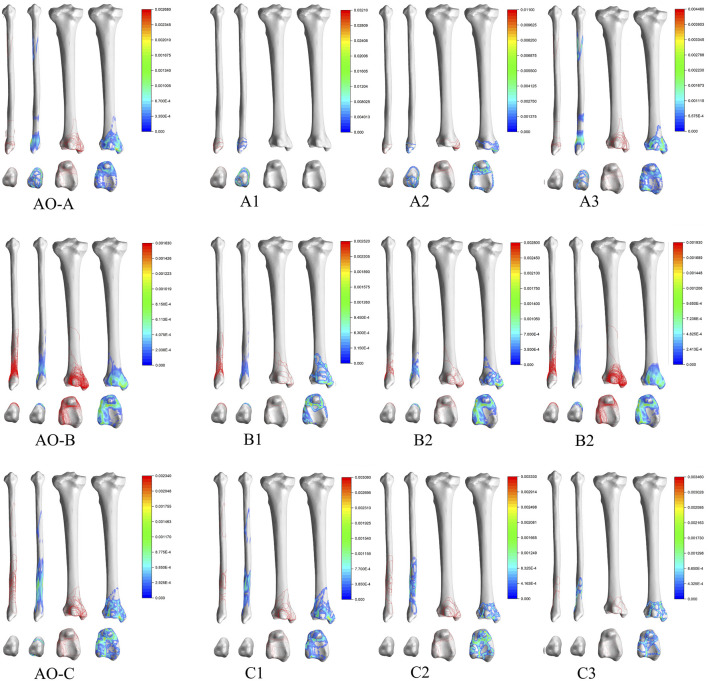
Distribution and hotpots of fractures by AO classification. The scale of heatmap represents relative frequency of fracture lines. From the blue area to the red area, the relative frequency gradually increases.

Type C: Type C3 fracture lines were distributed closer to the distal fibula than Type C2, and the portion of the fracture line on the tibia was similar to Type C2 (Figure 6).

## Discussion

As far as the published literature is concerned, although some studies have been conducted to perform fracture line mapping of ankle fractures using fracture line mapping techniques and heatmap techniques, they were limited to medial malleolus fracture or posterior malleolar fracture. This study is the first to apply 3D fracture line mapping techniques as well as heatmap techniques to analyze ankle fracture patterns.

We analyzed the 10 cases that could not be classified by AO classification and the 19 cases that could not be classified by LH classification. The fracture characteristics that could not be classified by LH or AO classification were summarized as follows: 1) All fractures were simple tibial fractures and no fibular fractures. 2) The most involved anatomic site was medial malleolus (14/19 of medial malleolus involvement in LH classification, 4/10 of medial malleolus involvement in AO classification) (Table 2).

**TABLE 2:** The method used for the mapping of ankle fracture.

Classification system	Medial malleolus fracture	Medial malleolus + posterior malleolus fracture	Chaput fracture	Posterior malleolus fracture	Total no. of unspecified fracture lines
LH	9	5	2	3	19
AO	0	4	2	4	10

* LH, Lauge-Hansen classification; AO, AO/OTA classification.

At present, scholars have made explorations related to the medial or posterior malleolus fracture line course and characteristics, based on which, they have proposed the corresponding classification. For example, Hraguchi et al. (Haraguchi et al., 2006) typed posterior malleolar fractures based on the morphology of posterior malleolar fractures in distal tibial cross-section under CT. They classified posterior malleolar fractures into three types, type I: the fracture fragment was wedge-shaped and involved the posterior external angle of the distal tibial articular surface; type II: the fracture line extended from the fibular notch of the distal tibia to the medial malleolar; type III: small shell type, where one or more small shell-shaped bone fragments were fractured at the posterior edge of the distal tibia. Mangnus et al. (Mangnus et al., 2015), on the other hand, qualitatively analyzed the morphology of posterior malleolar fractures based on the Haraguchi classification and divided the posterior malleolar fracture line into two categories: posterior external oblique (Haraguchi type I and III, both of which were fractures of different degrees caused by the same extensive force) and transverse (fracture line extending medially and involving the posterior malleolus of the medial malleolar; Haraguchi type II fracture).

For medial malleolus fracture, Herscovici et al. proposed a Herscovici classification in 2007 (Herscovici et al., 2007), which classified medial malleolus fracture into four categories A: avulsion fractures of the tip of the internal ankle, mostly involving the anterior malleolus and superficial deltoid ligament, while the deeper layer was more posterior; B: fracture line located between the level of the articular surface and the tip of the internal ankle; C: fracture line and articular surface were at the same level; D: the fracture line was vertical or obliquely directed inward and upward, mostly seen in the posterior rotational inversion type of injury. Less commonly, there was longitudinal instability.

From the reported literature, the fracture line alignment in the case of combined medial + lateral ankle involvement is consistent with the current definition of posterior pilon fracture. A specific type of posterior malleolar fracture with fracture line passing through the posterior malleolar node of the medial malleolar was reported by Pankovich et al. (Pankovich and Shivaram, 1979) in 1979. Ebraheim et al., 1990 named this type of fracture as a posterior collicular fractures of the medial malleolus (Ebraheim et al., 1990). By 2000 Hansen first named such type of fracture as posterior pilon fracture, which was characterized by a large fracture mass with one or more fracture masses and proximal displacement of the fractured mass to form a step, which may be accompanied by posterior or posterior-lateral subluxation of the talus. In 2004 Weber in his paper described 10 cases of posterior malleolar fracture characteristics of the posterior mound of the medial malleolar and proposed the double-contour or flake-fragment sign as the concept that the presence of such sign-on anteroposterior malleolar radiographs predicted extension of the posterior malleolar fracture mass to the posterior medial aspect, i.e., posterior malleolar involvement (Weber, 2004). According to the various existing medial malleolus fracture classifications, although it is possible to type such fractures, for example, type II in the Haraguchi classification and types III and IV in the Bartonícek classification fit the above definition of posterior pilon fracture. However, with the in-depth study of posterior malleolar fractures, it is now generally accepted that posterior pilon fracture is a special type of posterior malleolar fracture, and many scholars are trying to classify posterior pilon fractures. Klammer et al. (Klammer et al., 2013) divided posterior pilon fractures into three types based on clinical practice: type I accumulated the whole posterior malleolar, and the fractured mass was a long oblique shape with the base toward the posterior lateral side; type II fracture fragment included both posterior medial and posterior-lateral parts; type III was accompanied by anterior malleolus fracture of the medial malleolar. Yu et al. divided posterior pilon fractures into three types based on CT manifestations (Zhang and Yu, 2018), type I was a postero-lateral oblique type with a larger postero-lateral volkmann fracture mass; type II was a fracture line extending to the medial malleolar; type III fracture mass was divided into two parts: posterior medial and posterior lateral. Wang et al. (Lei et al., 2011) divided posterior pilon fractures into two types based on the location of the lateral malleolus fracture lines: type I fracture line was located in the lower tibiofibular union above and the medial posterior malleolar fracture fragment was not connected to the anterior malleolus fracture fragment of the medial malleolar; type II fracture line was located at the level of the inferior tibiofibular union and the medial posterior ankle fracture fragment was connected to the medial malleolar fracture fragment. In 2017, Zhang et al. proposed AGH classification (Zhang, 2017), type I posterior malleolar was a single intact bone block; type II was a posterior malleolar fracture fragment splits along the sagittal plane and was divided into two parts: posterior medial and posterior-lateral; type II b was a posterior malleolar fracture fragment split along the sagittal plane with a comminuted fracture of the posterior malleolar; type III a was fracture with a posterior malleolar fracture line accumulating the anterior malleolus of the medial malleolar and a complete fracture of the medial malleolar but without separation of the millia and posterior malleolus; and type III b was with a posterior malleolar fracture line accumulating the intermalleolar groove and an avulsion fracture of the anterior malleolus with separation of the anterior and posterior malleoli.

Although there have been many studies on classification of medial malleolus fracture, posterior malleolus fracture or posterior pilon fractures involving the entire coronal surface of the posterior malleolar, none of them can fully summarize all their characteristics, such as whether the fracture line is transverse or long oblique, whether it extends posteriorly, and medially to the posterior or anterior mound of the medial malleolar, and whether the fractured mass is 1-part, 2-part, or comminuted. The reason for the lack of uniformity in the understanding of the relevant classification is that the biomechanical mechanisms that lead to fractures involving the posterior malleolar fractures are not understood yet. Most of the currently available explanations for the mechanism of the fracture in question are derived from the analysis and extrapolation of the morphological features of the fracture in question on CT images (Bartoníček et al., 2015; Mason et al., 2017; Yi et al., 2018; Vosoughi et al., 2019). Posterior malleolar fractures have rarely been successfully simulated in biomechanical tests. Of the few relevant reports retrieved, Schaffer (Schaffer and Manoli, 1987), Michelson (Michelson et al., 1997), Stiehl (Stiehl et al., 1992), et al. failed to reproduce a posterior malleolar fractures in their respective experiments, except for a report by Haraguchi (Haraguchi and Armiger, 2020) in 2020 in which a fracture involving the entire posterior tibial margin, corresponding to Haraguchi type II, was reproduced, inferring that the mechanism of formation of this type of fracture as follows: As a result of axial loading of the talus, the posterior lower part of the tibiofibular ligament is torn leading to a posterior malleolar fractures with the formation of fracture fragments.

**FIGURE 7:**
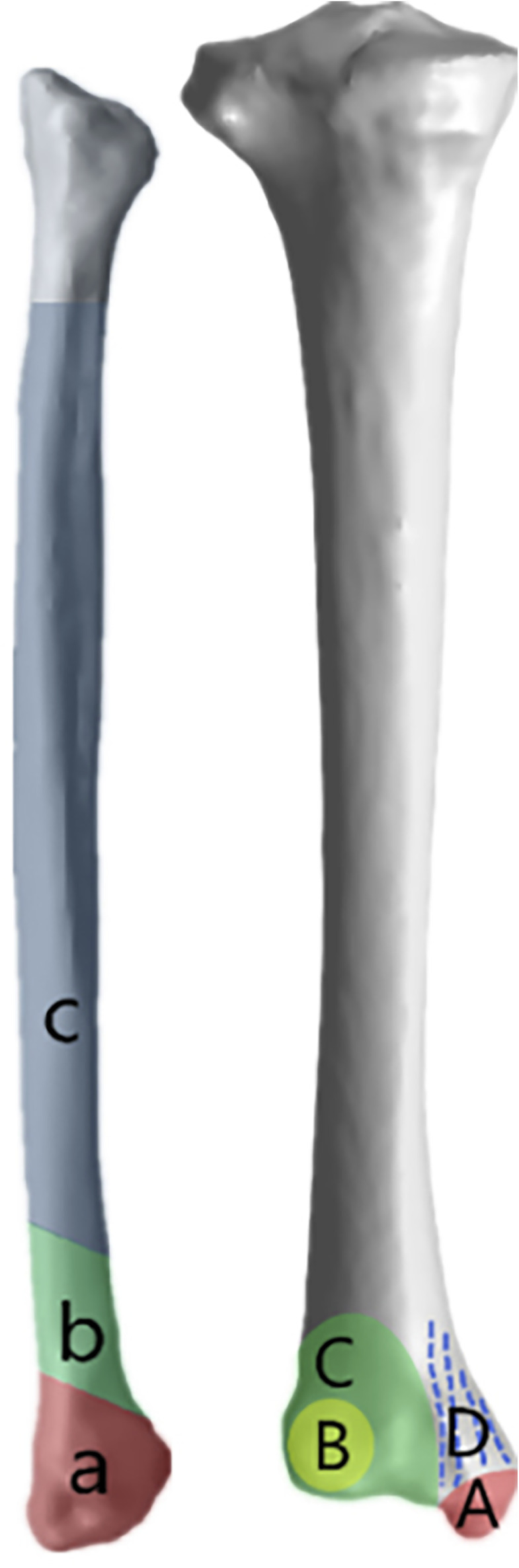
Different colored areas and corresponding letters on the surface of the bones represent the different types summarized in the Table 3.

The uncertainty of the fracture mechanism and the lack of uniformity in the classification have made it difficult to communicate the condition among physicians in the current clinical setting. The fracture lines and the distribution of hot spots on the heatmaps were summarized in Figures 2, 3, and the differences in the sites of different subtypes of fibula fractures in the existing Arbeitsgemeinschaftfür Osteosynthesefragen (AO) classification were also referred hereof, thus we tried to classify the fracture lines of the fibula and tibia into three categories and four categories, by analyzing the fracture map and heatmap, respectively (Table 3, Figure 7). In the area with the densest distribution of fracture lines (the green ring on the heatmap) in Figure 4, we defined it as category b (corresponding to the fibula fracture pattern of type B in the AO classification), then the fracture lines above this area we defined it as category c (corresponding to the fibula fracture pattern of type A in the AO classification), and the fracture lines below this area we defined it as a category a (corresponding to the fibula fracture pattern of type C in the AO classification). In Figure 3, the fracture line wrapped around the Volkmann’s node and the medial malleolus can be observed in a circular distribution area, and the corresponding green circular hot spot distribution can be observed on the heatmap, which is defined as type B and types A fractures, respectively. Outside the area of the fracture line encircling the Volkmann’s tuberosity (type B fracture line), which is the green circle encircling the Volkmann’s tuberosity on the heatmap, we can see the presence of an elliptical fracture line with a long axis parallel to the tibial stem, which exceeds the boundary of the type B fracture line. Similarly, outside of the circular fracture line encircling the medial ankle and the corresponding green circular hot spot, a distribution of fracture lines that travel vertically through the medial malleolus can be observed, and we define them as type C and D, respectively.

**TABLE 3:** A summary of fracture lines of fibula and tibia based on the 3D maps and heatmaps of fracture line distribution.

Anatomic sites	Classification	Description
Fracture lines of fibula	a	Located under the joint tibiofibular ligament and wrapped around the fibular head in a horizontal loop
	b	Located above the tibiofibular joint ligament, with the lowest point located at the anterior edge of the fibula, where the fibular head expands, and travels posteriorly and obliquely to wrap around the distal end of the fibula
	c	Located on the joint tibiofibular ligament and may wrap around the fibular stem in a circular or oblique fashion
Fracture lines of tibia	A	Over the medial malleolar joint surface, sub horizontally encircling the medial malleolar in a circular fashion
	B	surrounding the Volkmann’s tuberosity in a ring shape
	C	Similar to type B, but the annular fracture line was larger in diameter and extended upward from the lateral fibular notch of the tibia, crossing the anteromedial corner of the tibial fornix above, behind, and below Volkmann’s tuberosity, entering the subtalar articular surface, and running medially along the medial margin of the distal tibia to join the fibular notch fracture
	D	The fracture line run longitudinally from the subtalar articular surface up through the medial or posterior malleolus

Following this pattern of fracture line regional division, we reclassified the 228 patients (Table 4). A total of 32 combinations were identified, with fibula b being the most common of the single ankle fracture subtypes and fibula b + tibia AB being the most common of the trimalleolar fracture subtypes. Of course, such classification is based solely on the distribution of fracture lines, and it is only an attempt to map the ankle fracture lines in order to include fracture patterns that cannot be identified by Lauge-Hansen (LH) classification and AO classification and to differentiate and name them in order to facilitate communication between physicians about the condition.

**TABLE 4:** Fracture patterns organized by the method above.

Classification	No.(*n* = 228)	Classification	No.(*n* = 228)
0 + A	18	b + AD	5
0 + AB	4	b + ADB	1
0 + AC	4	b + B	24
0 + ACD	1	b + BD	3
0 + B	6	b + C	4
0 + C	4	b + CA	3
0 + D	5	b + CD	4
a+0	11	b + D	2
a+A	2	b + DA	1
a+AB	1	c+0	2
a+CD	1	c + A	2
a+D	4	c + AB	6
b+0	44	c + ADC	3
b + A	11	c + B	5
b + AB	30	c + BD	1
b + ABD	1	c + CD	2
b + AC	9	c + D	3
b + ACD	1		

* a, b, c represents the corresponding pattern of classification of fracture lines on fibula; A, B, C, D represents the corresponding pattern of classification of fracture lines on tibia.

The limitations of our study are as follows: Firstly, the number of patients was relatively small; Secondly, the proposed new fracture pattern in the conclusion is subjective, based on the summary of the distribution of ankle fracture lines and the reference to the existing fracture pattern of the fibula in the AO classification, thus lacking the biomechanical mechanism of the corresponding fracture pattern; Thirdly, due to anatomical variability (length of the tibiofibular, the cross-sectional aspect ratio of the distal tibiofibular, the size of the distal tibial articular surface, and the size of the anatomic structures such as the medial, lateral, and posterior ankle differ from patient to patient), some fracture reconstruction models did not exactly match the standard model of the tibiofibular; Fourthly, existing fracture line depiction techniques could only display the distribution of fracture lines on the surface of the tibiofibular and cannot display the distribution of fracture fractures within the cancellous mass.

## Conclusion

In this study, the 3D fracture line mapping technique was selected to describe the distribution of fracture lines throughout the ankle (not limited to the posterior or medial malleolar), with an attempt to classify the fibular and tibial fracture lines into three and four categories based on the distribution and density of lines to combine the two classifications to derive the fracture pattern of the ankle, respectively. And an attempt was made to classify 228 patients with ankle fractures using the above new method. The new classification method can include fracture types that cannot be identified by both AO and LH classification and is simple and convenient for physicians to communicate with each other about the patient’s condition. It is surely just an attempt to develop a uniform, comprehensive and accurate classification of ankle fractures. To truly develop a classification system that encompasses all ankle fracture types, a larger sample size of ankle fracture patients and an exploration of the biomechanical mechanisms of posterior ankle fractures are required.

## Data Availability

The raw data supporting the conclusions of this article will be made available by the authors, without undue reservation.
